# Coastal leatherback turtles reveal conservation hotspot

**DOI:** 10.1038/srep37851

**Published:** 2016-11-25

**Authors:** Nathan J. Robinson, Stephen J. Morreale, Ronel Nel, Frank V. Paladino

**Affiliations:** 1 The Leatherback Trust, Goldring-Gund Marine Biology Station, Playa Grande, Guanacaste, Costa Rica; 2 Department of Biology, Indiana University-Purdue University Fort Wayne, Fort Wayne, Indiana, USA; 3 Department of Natural Resources, Cornell University, Ithaca, New York, USA; 4 Department of Zoology, Nelson Mandela Metropolitan University, Port Elizabeth, South Africa

## Abstract

Previous studies have shown that the world’s largest reptile – the leatherback turtle *Dermochelys coriacea* – conducts flexible foraging migrations that can cover thousands of kilometres between nesting sites and distant foraging areas. The vast distances that may be travelled by migrating leatherback turtles have greatly complicated conservation efforts for this species worldwide. However, we demonstrate, using a combination of satellite telemetry and stable isotope analysis, that approximately half of the nesting leatherbacks from an important rookery in South Africa do not migrate to distant foraging areas, but rather, forage in the coastal waters of the nearby Mozambique Channel. Moreover, this coastal cohort appears to remain resident year-round in shallow waters (<50 m depth) in a relatively fixed area. Stable isotope analyses further indicate that the Mozambique Channel also hosts large numbers of loggerhead turtles *Caretta caretta*. The rare presence of a resident coastal aggregation of leatherback turtles not only presents a unique opportunity for conservation, but alongside the presence of loggerhead turtles and other endangered marine megafauna in the Mozambique Channel, highlights the importance of this area as a marine biodiversity hotspot.

Establishing the movement patterns of free-ranging animals is imperative to understanding their behaviour and ecology, and often is central to designing effective conservation strategies^1^. Furthermore, animal movements can provide insights into the characteristics of the land- or sea-scape that the animal is travelling through, such as the distribution of prey or thermal habitats^2^,^3^,^4^. This is especially true for animals that undergo extensive migrations throughout their lives, such as a wide range of marine mammals, fish, and sea birds. Sea turtles are also well known for their long-distance migrations, with arguably the longest undertaken by the leatherback turtle *Dermochelys coriacea*^5^.

Leatherback turtles can travel thousands of kilometres between tropical nesting beaches and foraging areas, which are often in remote, open-ocean waters^6^,^7^,^8^,^9^. These foraging areas usually coincide with regions that host large abundance of gelatinous zooplankton^4^ – the obligate prey of leatherback turtles. The size of these foraging areas can extend from localized regions, to entire ocean basins^7^,^8^. In addition, leatherback turtles may feed opportunistically en route to these foraging areas and, due to the spatiotemporally ephemeral nature of the gelatinous zooplankton blooms in the open-ocean^10^,^11^, this means that the migratory pathways of leatherback turtles generally have a flexible, meandering appearance^12^.

Much like leatherback turtles, loggerhead turtles *Caretta caretta* have been observed conducting meandering foraging movements in the open-ocean^13^,^14^. Here they feed opportunistically on a variety of hard-shelled and gelatinous invertebrates^15^. However, even loggerhead turtles from the same nesting cohorts, have also been reported to migrate to coastal habitats^16^,^17^, where they feed on aggregations of benthic invertebrates^18^. The movements of these coastally foraging loggerhead turtles tend to be far more direct than their oceanic counterparts^19^ and many often closely follow the coastal line^20^. Moreover, these coastal migrations tend to be shorter and the shallow-water foraging areas are often relatively localized and consistent between years^21^,^22^.

Arguably, the most common technique for tracking the movements of highly mobile marine megafauna is satellite telemetry^23^. Modern animal-borne satellite transmitters are capable of relaying locations of an animal on fine spatial and temporal scales over periods of time that, in some cases, may extend up to years. Furthermore, important insights gained by satellite telemetry can be augmented with other contemporary tools, such as stable isotope analysis. Stable isotope analysis is a useful tool for animal tracking, as individuals that forage on different foods, or in different locations, tend to have different isotopic signatures^24^. By combining stable isotope analysis with satellite telemetry, it is possible to connect the stable isotopic values of individuals to those of their foraging areas. Once the stable isotopic signatures of different foraging areas are mapped out, it is then possible to infer an animal’s foraging area from stable isotope analysis alone. As stable isotope analysis is relatively cheap, it is a useful tool for scaling-up the inferences that can be gained from a relatively small number of satellite telemetry devices.

Here, we re-examined the breadth and diversity of movement patterns of leatherback and loggerhead turtles that nest in iSimangaliso Wetland Park, South Africa – an important nesting rookery in the Indian Ocean - using a combination of satellite telemetry and stable isotope analysis. First, we deployed 16 satellite transmitters onto post-nesting leatherback turtles between 2011 and 2013. Because female leatherback turtles lay up to several nests in a single season, we used a portable ultrasound device to select turtles that were depleted of enlarged ovarian follicles, and thus were poised to begin their post-nesting migrations. The selected 16 leatherback turtles were tracked by satellite as they travelled distances up to 10,000 km from the nesting areas; the mean tracking duration was 111.5 ± 41.3 days. Second, we collected skin samples from 96 leatherback, including all individuals with satellite transmitters, and 120 loggerhead turtles at the same nesting locations. The skin samples were used for δ^13^C and δ^15^N stable isotope analysis.

## Results

**Figure 1:**
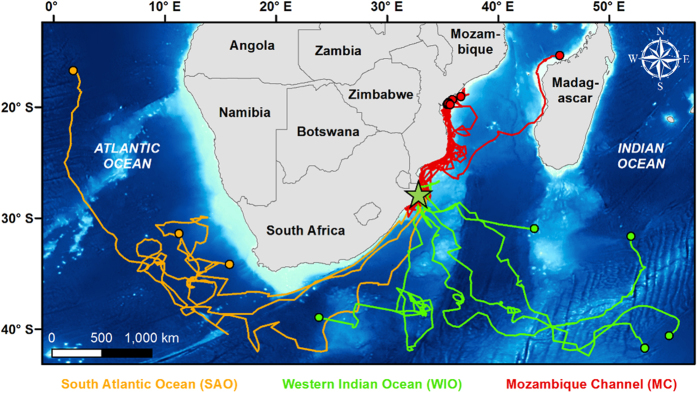
Movements of 16 post-nesting leatherback turtles tracked from the iSimangaliso Wetland Park, South Africa (green star) using tethered satellite transmitters between 2011 and 2013. Three migratory behaviours were observed, with individuals travelling distances of up to 10,000 km towards the South Atlantic Ocean (orange lines), the Western Indian Ocean (green lines), or the Mozambique Channel (red lines). The final relayed location for each turtle is signified by a coloured circle. Map was created using ArcGIS v. 10.3 (http://www.esri.com/software/arcgis).

**Figure 2:**
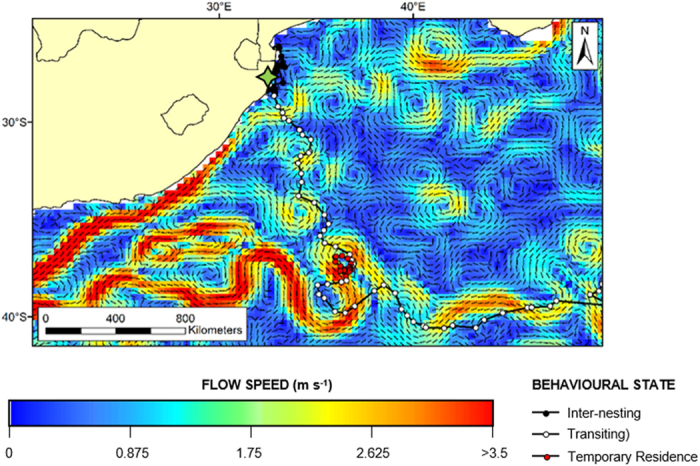
Daily locations (circles) of a single leatherback turtle that was tracked by satellite from the iSimangaliso Wetland Park, South Africa as it conducted its post-nesting migrations into the open-ocean waters of the Western Indian Ocean. Black circles represent inter-nesting behaviour, white circles represent transiting behaviour, and red circles represent a period of temporary residence within a mesoscale eddy. The track is overlaid onto ocean current data for the 5-day period between 1/4/12 and 6/4/12. Map was created using ArcGIS v. 10.3 (http://www.esri.com/software/arcgis).

Half (n = 8) of the satellite-tracked leatherback turtles in this study migrated southwards of the nesting area, often following the prevailing Agulhas Current down the east coast of South Africa. Upon reaching, the southern tip of the continent, these turtles began to conduct meandering movements in either the Western Indian Ocean, or the South Atlantic Ocean (Fig. 1). Many of these migrations were punctuated by short periods of residence (<15 days) in locations that generally coincided with the nearby presence of meso-scale eddies (Fig. 2), and after leaving the area of the eddies, the turtles continued as before with similarly nomadic movements. These flexible migratory patterns are very similar to those previously observed in prior tracking studies on leatherback turtles in the region^25^,^26^.

**Figure 3:**
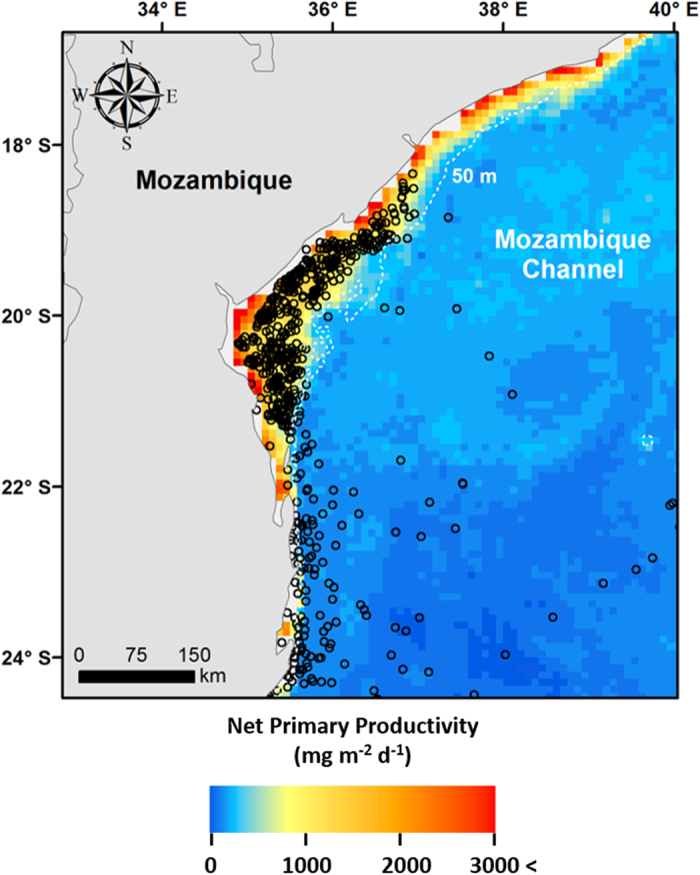
Daily locations (hollow circles) of 7 post-nesting leatherback turtles that were tracked by satellite from the iSimangaliso Wetland Park, South Africa to foraging areas in the Sofala Banks, Mozambique. These 7 individuals were tracked for between 91 and 209 days after their final nest. Turtle locations are overlaid onto a map of Net Primary Productivity for the month of Apr 2012. Turtles took up residence in the shallow waters of the Sofala Banks that are delineated by the 50 m isobaths (dashed white line). Coastal foraging is far more common in the population than previously acknowledged, and could be more prominent globally than previously considered. Map was created using ArcGIS v. 10.3 (http://www.esri.com/software/arcgis).

In marked contrast, the other half (n = 8) of the satellite-tracked leatherback turtles migrated a relatively short distance (~500 km) northwards into the Mozambique Channel. Seven of these eight individuals took up residence in the waters of the Sofala Banks, while the eighth migrated across the channel to coastal waters of Madagascar. In these shallow (<50 m depth) coastal waters, individuals reduced their speed to less than 5 km d^−1^. For the remainder of the tracking duration, which in one case extended to 209 days, the seven turtles that migrated to the Sofala Banks remained within a narrow swath along coastal waters less than 100 km wide. Notably, both areas of residence on either side of the Mozambique Channel were characterized by high Net Primary Productivity (NPP) (Fig. 3). Moreover, NPP remains consistently elevated in these waters year-round, and sea surface temperatures always remain habitable for leatherback turtles at >20 °C.

**Figure 4:**
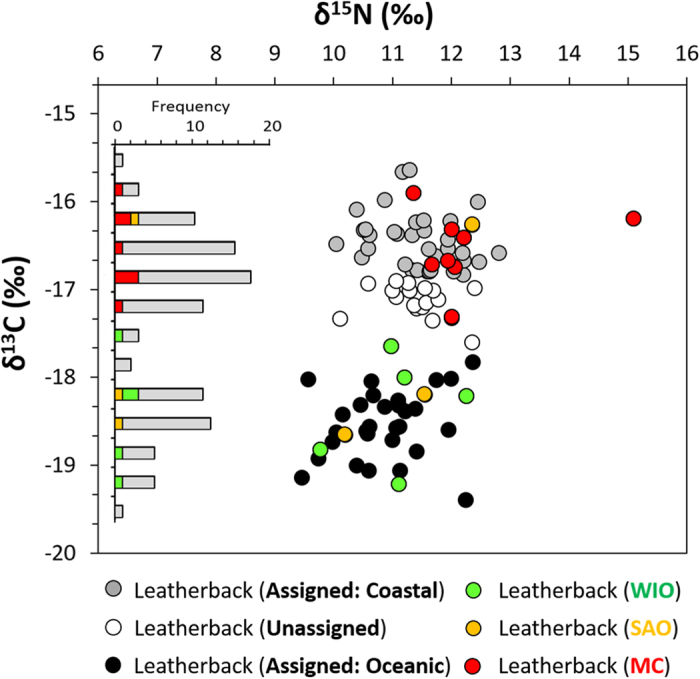
Stable isotope values from skin samples collected from nesting leatherback turtles within the iSimangaliso Wetland Park, South Africa between 2011 and 2013. Coloured circles represent individuals that were tracked using satellite telemetry into oceanic foraging areas in the Western Indian Ocean (WIO, green circles) or the South Atlantic Ocean (SAO, orange circles), as well as coastal foraging areas in the Mozambique Channel (MC, red circles). Discriminant function analysis was used to assign foraging areas for turtles that were not tracked by satellite. Turtles with >80% probability of group membership were designated as either coastal (gray circles) or oceanic individuals (black circles). Individuals with <80% probability of group membership were left unassigned (clear circles). Leatherback turtles tracked to coastal foraging habitats in the Mozambique Channel had distinct stable isotopic values from those tracked to oceanic foraging habitats in the Western Indian or South Atlantic Ocean.

The combination of both oceanic- and coastal-foraging strategies in this population of leatherback turtles, as observed through satellite telemetry, was further reinforced by our subsequent stable isotope analyses. Skin samples from the 96 leatherback turtles had δ^13^C values ranging from −19.1 to −15.2‰, with a distinct bi-modal distribution and an apparent delineation between the two clusters at approximately −17.5‰ (Fig. 4). Importantly, the stable isotopic signatures of the 8 leatherback turtles that were tracked to coastal foraging areas in the Mozambique Channel all fell into the high δ^13^C cluster, whereas all but one of the turtles tracked to oceanic foraging areas fell in the low δ^13^C cluster. The δ^15^N values had a unimodal distribution ranging from between 9.5 and 15.1‰, and did not show separation between coastal and oceanic turtles. Overall, the stable isotopic values for δ^13^C and δ^15^N of these oceanic and coastal groups were significantly different (MANOVA: F_1,15_ = 9.51, p = 0.003).

Using the stable isotope values of the satellite-tracked leatherback turtles, we calculated a discriminant function to predict whether the remaining 81 individuals where previously foraging in either oceanic or coastal habitats. To test the robustness of the discriminant function, we applied it to the satellite-tracked turtles first, and it was able to correctly assign foraging areas for all but two of the satellite-tracked individuals (88% assigned correctly). The robustness of the discriminant function analysis was further tested using a jackknife cross-validation method that performed just as well as the original model (88% assigned correctly). The discriminant function was then applied to all individuals that were not tracked by satellite, assigning 61 out of 81 turtles to either oceanic or coastal foraging habitats. Of the 61 turtles with assigned foraging areas, 33 (41%) were designated as coastal foragers while 29 (36%) were designated as oceanic foragers. The remaining 19 (23%) individuals could not be assigned to coastal or oceanic habitats with >80% probability.

**Figure 5:**
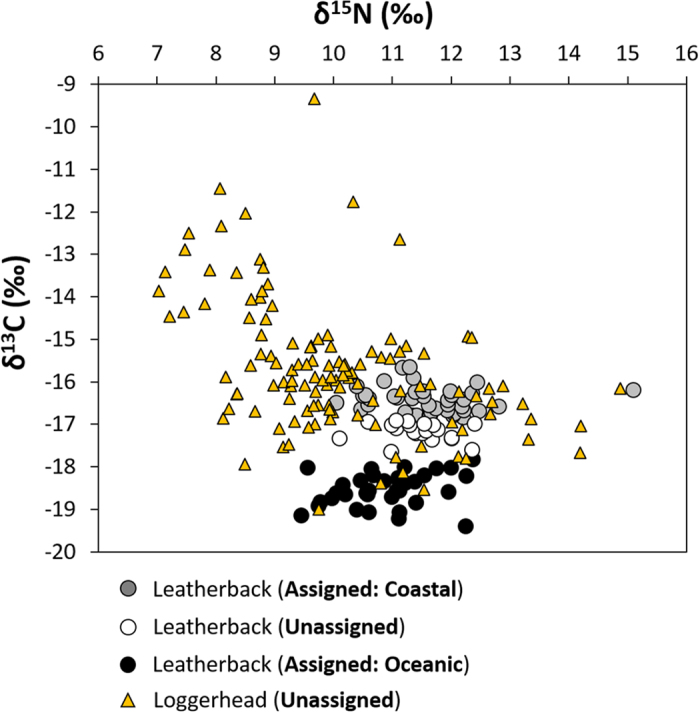
Stable isotope values from skin samples collected from nesting loggerhead and leatherback turtles within the iSimangaliso Wetland Park, South Africa between 2011 and 2013. Coloured circles represent individuals that were tracked using satellite telemetry into oceanic foraging areas in the Western Indian Ocean (WIO, green circles) or the South Atlantic Ocean (SAO, orange circles), as well as coastal foraging areas in the Mozambique Channel (MC, red circles). Loggerhead turtles (blue triangles), have a broader range of δ^13^C values than leatherback turtles (circles). There was a large overlap in δ^13^C between loggerhead turtles and leatherback turtles assigned to coastal foraging areas (grey circles) using a discriminant function analysis.

While collecting the leatherback turtle skin samples, we also collected skin samples from 120 sympatrically nesting loggerhead turtles, Although the loggerhead turtles had a much broader range of both δ^13^C and δ^15^N values, from −19.0 to −9.4‰ and 7.0 to 14.9‰ respectively, the values overlapped with those of the leatherback turtles (Fig. 5). Notably, the majority (92%) of the loggerhead turtle samples had values of δ^13^C below the putative −17.5‰ coastal discriminant function threshold that was calculated for the leatherback turtles.

## Discussion

This is the first published study to track leatherback turtles by satellite telemetry into the Mozambique Channel. Furthermore, considering that the Sofala Banks retains perpetually elevated NPP levels and SSTs that are habitable for leatherback turtles, we predict that this region hosts foraging leatherback turtles year round, as well as abundant quantities of gelatinous zooplankton prey. Ecologically, this discovery is particularly interesting as leatherback turtles are often considered open-ocean specialists and although visual observations and satellite telemetry have confirmed that leatherback turtles can be found in coastal waters, their presence near shore is usually temporary and often ascribed to ephemeral blooms in jellyfish abundance^27^,^28^,^29^. The only other location where leatherback turtles are known to be resident in coastal foraging areas is in the Western Pacific, specifically around Indonesia, in the waters of the South China Sea, and along the west coast of Australia^8^. In our satellite tracking study, half of the post-nesting turtles migrated up to thousands of kilometres to distant waters of the Indian and Atlantic Oceans, while the other half travelled minimal distances to become resident in nearby coastal waters.

In addition to coastally foraging leatherback turtles observed in this study, other individuals were observed conducting more typical flexible foraging movements in the open ocean. The dichotomy of migratory strategies, as revealed through satellite telemetry, was further substantiated by stable isotope analyses. Informatively, all the leatherback turtles that were satellite-tracked to coastal habitats fell into the high δ^13^C cluster, while all but one individual tracked into oceanic habitats fell into the low δ^13^C cluster. Applying the discriminant function derived from this analysis, we conservatively estimate that 41% of the 97 leatherback turtles sampled forage in coastal environments. This was slightly higher than the 36% identified as foraging in oceanic environments. Thus, it appears that the Mozambique Channel is likely the most important foraging habitat for the leatherback turtles that nest in South Africa. In addition, we did not track any male or juvenile leatherback turtles in this study, but we hypothesize that the Mozambique Channel is likely to be a similarly, if not more, important foraging area at least for males of this species. Indeed, tracking studies on sea turtles have indicated that male turtles utilize similar foraging areas as female turtles^30^, and they tend more frequently to utilize foraging habitats closer to the nesting area than do the females^31^,^32^.

The mixed migratory strategies observed here were different than previous satellite tracking studies on leatherback turtles in the region, which only reported individuals migrating into the open-ocean and not to the Mozambique Channel^25^,^26^. One explanation could be due to the small sample size of those studies. However, given that we conservatively estimate that 41% of this population are coastal foragers through stable isotope analysis, then the probability that none of the nine previously tracked leatherback turtles migrated to the Mozambique Channel is nominal (<0.1%). It is also unlikely that the lack of coastal foragers in the previous studies could be due to inter-annual variation in oceanographic conditions, as both our study and the previous satellite tracking studies at this location, were conducted over multiple years. Moreover, the prevailing flow of the Agulhas Current, which is reported to have accounted for a large proportion of the southerly movements of these previously tracked leatherback turtles^25^,^33^, is remarkably consistent between years^34^. Alternatively, we propose that the differences may be due to the different modes of attachment of the satellite transmitters. The previous studies utilized harnesses^35^ to attach the transmitters, which have been linked in some cases to behavioural changes in the movement patterns of migrating leatherback turtles^36^, and have been shown to potentially increase hydrodynamic drag to swimming leatherback turtle by over 90%^37^. It is therefore possible that the low-drag hydrodynamic tether attachment used in this study^38^ could be more reflective of the full breadth of migratory movements of these animals.

Dichotomous migratory strategies, as observed in the leatherback turtles, are common for loggerhead turtles^17^,^39^,^40^. Our stable isotope analysis suggests such dichotomous behaviour also exists for the loggerhead turtles that nest in South Africa; however, using the same coastal/oceanic threshold for loggerhead turtles as calculated for the stable isotope values for leatherback turtles, the vast majority (92%) would be assigned as coastal foragers. Such inter-species comparisons using δ^13^C values are generally valid as δ^13^C does not change along trophic interactions, and generally remains the same for organisms feeding in a similar area^41^. The pattern of higher δ^13^C values in coastal animals relative to oceanic animals has also been observed elsewhere in marine mammals^42^, seabirds^43^, and marine invertebrates^44^. Although this indicates that loggerhead turtles are feeding in coastal habitats, it does not confirm that they are feeding in the same location as the leatherback turtles. Nevertheless, in conjunction with previous satellite tracking studies^45^,^46^, we can assume that these loggerhead turtles are also feeding in the wider Mozambique Channel region.

In contrast to δ^13^C, values of δ^15^N are often enriched by 3 to 4‰ per trophic level^47^. As such, δ^15^N values can be an informative indicator of the trophic level at which an animal forages^48^. The small range of δ^15^N values for leatherback turtles observed in this study (3.4‰ when excluding a single individual of 15.1‰) suggests that this species was foraging on the same trophic level, whether in coastal or oceanic habitats. Indeed, leatherback turtles appear to be obligate gelatinous zooplanktivores and, even though jellyfish can vary greatly in δ^15^N values^49^, leatherback turtles likely target specific, and often larger, jellyfish species^50^. Conversely, loggerhead turtles exhibit a breadth of dietary preferences, including a variety of invertebrate and fish species^18^. This appears to have been reflected in the wider range of δ^15^N values among the 120 loggerheads sampled.

From a conservation perspective, considering that leatherback and loggerhead turtles are feeding on different trophic levels, and a single leatherback can consume in excess of 100 kg wet mass of jellyfish per day^51^, the Mozambique Channel must presumably host large abundances and a wide diversity of food. Indeed, with high levels of primary productivity, nutrient-rich river mouths and nearby areas of coastal upwelling and meso-scale eddies, these coastal waters also provide fertile foraging areas for a wide range of megafauna. For example, the Mozambique Channel hosts the largest populations of dugongs *Dugong dugon* in East Africa^52^, as well as substantial populations of whale sharks *Rhincodon typus*^53^, giant *Manta birostris* and reef *M. alfredi* manta rays^53^, and numerous cetaceans^54^.

Simultaneously, the region also hosts large abundances of many commercially important species, such as the Indian prawn *Penaeus indicus*, and this has led to many conservation issues^55^. The shrimp-trawl fishery in the Mozambique Channel is one of the most profitable industries in the country, but it often incurs substantial by-catch of sea turtles, and many other species^56^. Mortality associated with this by-catch may even partly explain why the population of leatherback turtles nesting in South Africa is not recovering after the comprehensive protection of its nesting habitats over many years^57^.

Yet, the discovery of leatherback turtles congregating in coastal habitats, alongside loggerhead turtles, could also provide unique opportunities for conservation. Previously, the presumed oceanic habits of leatherback turtles has created a challenge for conservation management due to the vast geographic scope and range of factors that could pose a threat to populations at sea. However, at the Sofala Banks and potentially other regions where sea turtles are concentrated in coastal habitats, it may be much easier to implement spatially-explicit management strategies for protecting these populations than it is for leatherback turtles elsewhere.

Delineating specific key habitats might also provide opportunities for otherwise complex multinational conservation plans. Uniquely, the leatherback and loggerhead turtles foraging in these waters occupy a relatively small and fixed foraging area within the sole EEZ of Mozambique. Indeed, identifying and safeguarding coastal habitats, in addition to existing measures that currently protect nearby nesting beaches, might prove to be highly beneficial for endangered populations.

## Methods

### Study Site

The iSimangaliso Wetland Park is located along the northeast corner of South Africa (28°0′ S, 32°30′ E). The shoreline of the park is approximately 280 km long and is characterized by a series of 5–15 km stretches of sandy beaches separated by rocky headlands. Each year from October until February, female leatherback and loggerhead turtles come ashore at night to nest^57^.

Between 2011 and 2013, we conducted nightly patrols of the northern most 56 km of the park’s coastline to search for nesting turtles. When nesting leatherback and loggerhead turtles were encountered we applied individually numbered metal and passive integrated transponder (PIT) tags and collected skin samples. For leatherback turtles, we also attached satellite transmitters when possible. Nesting turtles were not approached until egg laying had commenced to minimize the potential of interrupting the nesting process.

All field work was approved by, and conducted with the knowledge of, the iSimangaliso Wetland Park Authority and the Department of Environmental Affairs, South Africa. The satellite tagging and skin sample collecting methods were performed in accordance with the approved guidelines of the Animal Care and Use Committee of Purdue University, USA. Turtle skin samples were imported into the US under CITES permits (#12U589757A/9).

### Satellite transmitters

Satellite transmitters (MK10-PAT, Wildlife Computers) were deployed on 20 nesting leatherback turtles using a low-drag tethering technique. The transmitters, tethered by lanyard and anchored to the pygal process that extends to the rear of the carapace, were designed to be towed behind the swimming animal, traveling in its slipstream. For full details on the transmitter attachment method see Supplementary Information.

In this study, we aimed to track the post-nesting migrations of leatherback turtles. As leatherback turtles lay multiple clutches in a single nesting season, we used ultrasonography to gauge whether or not a turtle had laid all of its clutches for the season and was thus about to begin its post-nesting migration. Using a Sonosite 180 Plus real-time portable ultrasound unit, we scanned the inguinal cavity of leatherback turtles to examine the turtle ovaries for evidence of vitellogenic follicles^58^,^59^. If less than five vitellogenic follicles were present per ovary, we inferred that the turtle had laid its final clutch for that season and was thus a suitable candidate for the attachment of a satellite transmitter. Satellite transmitters were only attached to turtles that appeared to be in good health and with no evident injuries.

### Tissue sampling and preparation

Skin samples were collected from a total of 96 leatherback turtles (including all satellite tracked leatherback turtles) along with 120 loggerhead turtles. A total of 27 leatherback turtles were sampled more than once, while none of the loggerhead turtles were sampled multiple times. When an individual was sampled more than once, we used the mean values for δ^13^C and δ^15^N in all subsequent analyses. Skin samples were collected in preference to other superficial tissues (e.g. blood), because the isotopic turnover of skin in large reptiles is on the scale of months to years^60^,^61^. Thus, it should reflect foraging conditions at the previous feeding areas, and not vary significantly over the nesting season.

Skin samples were collected using a sterile 6-mm biopsy punch from the medial edge of the front or rear flipper, avoiding any previous scar tissue. After the skin sample was removed, the area was sterilized using antiseptic spray (oxytetracycline). The skin sample was immediately stored in 95% undenatured ethyl ethanol and kept at room temperature during transport and storage. In the lab, the upper layer of the skin (stratum corneum; subsequently referred to only as skin) was separated from the underlying tissue using a scalpel. The remaining skin was rinsed with deionized water and diced into 10 to 20 pieces. The diced samples were dried for a minimum of 6 h using a rotary evaporator. The dried samples were weighed using a microbalance and between 0.3 and 1.0 mg of the sample was packed into tin capsules for mass spectrometry.

### Stable isotope analysis

Stable isotope analyses were conducted at the Purdue Stable Isotope Facility, housed in the Department of Earth, Atmospheric, and Planetary Sciences at Purdue University, USA. The ratio of ^13^C to ^12^C and ^15^N to ^14^N in each sample was determined using a Carlo Erba 1108 Elemental Analyser, coupled with a Sercon 20–22 Continuous Flow Isotope Ratio Mass Spectrometer. Stable isotope values were expressed in delta (δ) notation relative to universal standards in parts per thousand (‰) using the following equation:


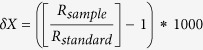


X refers to ^13^C or ^15^N. R_sample_ is the ratio of heavier to lighter isotopes of the appropriate element in the sample, while R_standard_ is the ratio of heavier to lighter isotopes of the standard. The standard for δ^13^C is Pee Dee Belemnite and for δ^15^N is atmospheric nitrogen. Analyses were calibrated, to ensure reproducibility, using replicates of Peach Leaf standards (NIST1547) with standard deviations (σ) of δ^13^C being ≤0.2‰, and δ^15^N being ≤0.35‰. We employed a post-hoc correction factor to account for lipids in the samples^62^. To assess stable isotope variation between skin samples, 27 samples were chosen at random and run in duplicate. The standard deviation (σ) between duplicate samples in δ^13^C was 0.37‰, and for δ^15^N was 0.55‰.

### Analysis of movement data

Locations from the satellite transmitters were obtained via the Argos Satellite System (Maryland, USA). These data were filtered using a maximum speed filter of 240 km d^−1^ and only the most accurate location (based on Location Class) per day was retained. For days when no data were available, a Bayesian state-space model^63^ was used to estimate daily locations based on the speed-filtered data. The state-space model generated daily position estimates for each turtle, and was run with 2 chains for 30,000 Markov Chain Monte Carlo samples with a 10,000 burn in (thin = 5). Only 10% of the final locations used modelled data, and we preferentially used the raw data instead of the state-space modelled data, as the modelled data occasionally provided seemingly spurious results.

The satellite telemetry data were overlaid onto spatially referenced bathymetry and Net Primary Productivity (NPP) datasets. Bathymetry data at a spatial resolution of 0.017° were provided by the global relief model, ETOPO1, available at the National Geographic Data Center, USA (http://www.ngdc.noaa.gov/mgg/global/). Monthly composites of NPP at a spatial resolution of 0.083° were provided by the Epperly-VPGM model available at the Oregon State University Ocean Productivity Page (http://www.science.oregonstate.edu/ocean.productivity/index.php). 5-day composites of ocean currents at a spatial resolution of 0.333° were provided by the Ocean Surface Current Analysis Real-time (OSCAR) (http://podaac-www.jpl.nasa.gov/dataset/OSCAR_L4_OC_third.deg). Maps were created using ArcGIS v. 10.3 (https://www.arcgis.com/).

### Statistical analyses

To test if leatherback turtles tracked by satellite to different foraging areas exhibited distinct stable isotope values, we used a Multivariate Analysis of Variance (MANOVA) with a Pillai’s trace test to compare the values of δ^13^C and δ^15^N from turtles with separate putative foraging areas. Data were tested for normality and homogeneity using Kolmogorov-Smirnov and Levene’s test respectively. If the isotopic values of turtles from different foraging areas were statistically different, it would provide evidence that these animals display foraging site fidelity between nesting seasons. More importantly, it would also infer that stable isotope analysis is a suitable tool for inferring foraging areas for sea turtles in the region.

Once our results indicated that stable isotope analysis could be used to infer if leatherback turtles were foraging in either coastal or oceanic habitats, we employed a Linear Discriminant Function Analysis to determine the foraging habitats of the turtles that were not tracked by satellite. We used the δ^13^C and δ^15^N ratios of the 16 satellite-tracked leatherback turtles with known foraging areas as a training data set (using equal weighted priors) to define the discriminant functions. The derived discriminant functions were used to determine the probability that each leatherback turtle that was not tracked belonged to a specific foraging area. If the probability was >80%, an individual was assigned to that foraging area. To test the accuracy of our assignments, we used jackknife (leave-one-out) cross-validation. In this method, each of the satellite-tracked turtles is removed from the training dataset and then classified to a foraging area using the discriminant functions derived from the remaining satellite tracked turtles. Data were analysed using the program R (R Development Core Team 2011) with an α level of 0.05.

## Additional Information

**How to cite this article**: Robinson, N. J. *et al.* Coastal leatherback turtles reveal conservation hotspot. *Sci. Rep.* **6**, 37851; doi: 10.1038/srep37851 (2016).

**Publisher's note:** Springer Nature remains neutral with regard to jurisdictional claims in published maps and institutional affiliations.

## Supplementary Material

Supplementary Information
