# Supplementary material for: Coastal leatherback turtles reveal conservation hotspot
Source: Sci Rep. 2016 Nov 25;6:37851. doi: 10.1038/srep37851 (PMC5122952; doi:10.1038/srep37851)
Supplement: Supplementary Information [file srep37851-s1.doc]

**Title:**

Coastal leatherback turtles reveal conservation hotspot

**Authors:**

Nathan J. Robinson^1,2*^, Stephen J. Morreale^3^, Ronel Nel^4^, Frank V. Paladino^1,2^

**Affiliations:**

^1^The Leatherback Trust, Goldring-Gund Marine Biology Station, Playa Grande, Guanacaste, Costa Rica

^2^Department of Biology, Indiana University-Purdue University Fort Wayne, Fort Wayne, Indiana, U.S.A

^3^Department of Natural Resources, Cornell University, Ithaca, New York, USA

^4^Department of Zoology, Nelson Mandela Metropolitan University, Port Elizabeth, South Africa

*Corresponding author: [nathan@leatherback.org](mailto:nathan@leatherback.org)

**Emails:**

Nathan J. Robinson – nathan@leatherback.org

Stephen J. Morreale - sjm11@cornell.edu

Ronel Nel – ronel.nel@nmmu.ac.za

Frank V. Paladino – paladino@ipfw.edu

**SUPPLEMENTARY INFORMATION**

*Methodology for attaching towable devices to sea turtles*

The tethering method used to attach the transmitter (Supplementary Figure 1) can be divided into a six major steps (Supplementary Figure 2).

(Step 1) An electric drill with a sterilized drill bit was used to create a 5 mm diameter incision 20 to 30 mm from posterior edge of the pygal process. The incision was immediately treated with spray antiseptic.

(Step 2) The ‘needle’ – a pre-made length of surgical tubing of 5 mm diameter and walls of 1 mm thickness attached to 45 kg (100 lb) fishing line using an equivalent strength crimp – was pulled through the incision, leaving some surgical tubing protruding both above and below the carapace.

(Step 3) The protruding surgical tubing was cut flush with the carapace.

(Step 4) A length of 180 kg (400 lb) flexible fishing line approximately 1.25 m long was threaded halfway through the incision. To reduce friction between the surgical tubing and the fishing line, the fishing line was coated with a water-based lubricant. The fishing line extending below the carapace was looped through the ‘lower button’ – a delrin cylinder 40 mm in diameter and 15 mm in height with an upside-down Y-shaped hole in the centre. The fishing line was then re-thread back through the surgical tubing, reapplying lubricant if needed.

(Step 5) On the dorsal-side of the carapace, both ends of the fishing line were passed through the ‘upper button’ – a delrin cylinder 20 mm in diameter and 10 mm in height with a straight hole in the middle. Both ends of the fishing line were pulled taught and crimped directly above the upper button using 180 kg (400 lb) crimps.

(Step 6) The longest free-length of fishing line was fastened using a crimp to the swivel on the transmitter’s ‘lanyard’ at a length of between 25 and 30 cm. The ‘lanyard’ was formed of a length of fishing line approximately 15 cm long that was attached at opposing ends to a 180 kg (400 lb) swivel and the Mk10 PAT with 180 kg (400 lb) crimps. As a result, the total length of the tether was between 40 and 55 cm from the upper button to the base of the transmitter. The exact length was decided in the field, ensuring that the Mk10 PAT could not be reached by the front flippers but would be able to reach the surface when the turtles emerged to breathe. When it was confirmed that the transmitter was securely attached to the turtle, all the excess fishing line was trimmed to the base of the crimps using wire cutters.

*Recovering and reattaching a new transmitter*

If a turtle with an attached satellite transmitter was encounter during a subsequent nesting event the transmitter was recovered and a new device was deployed. To remove a transmitter, the fishing line beneath the lower button was cut and the tether was pulled free. To deploy a new transmitter required only steps 4 to 6.


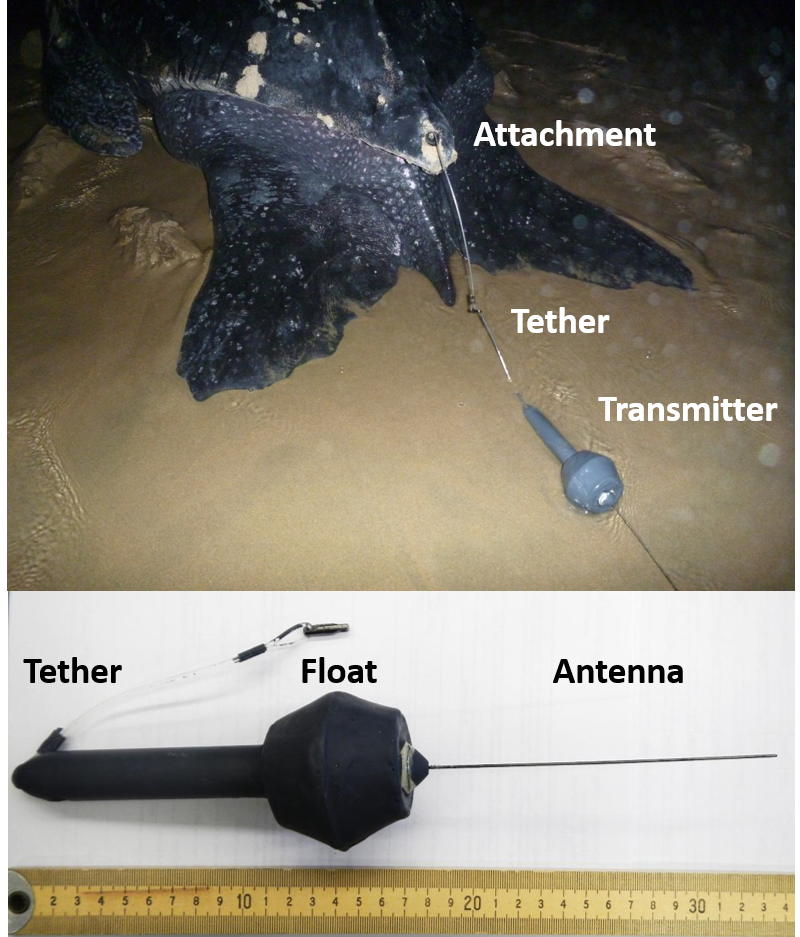


**Supplementary Figure 1.** Above - Image to show the attachment of a towed satellite transmitter to a leatherback turtles *Dermochelys coriacea* using the tethering technique. Below – Close up of the MK10-PAT transmitter for scale.

**
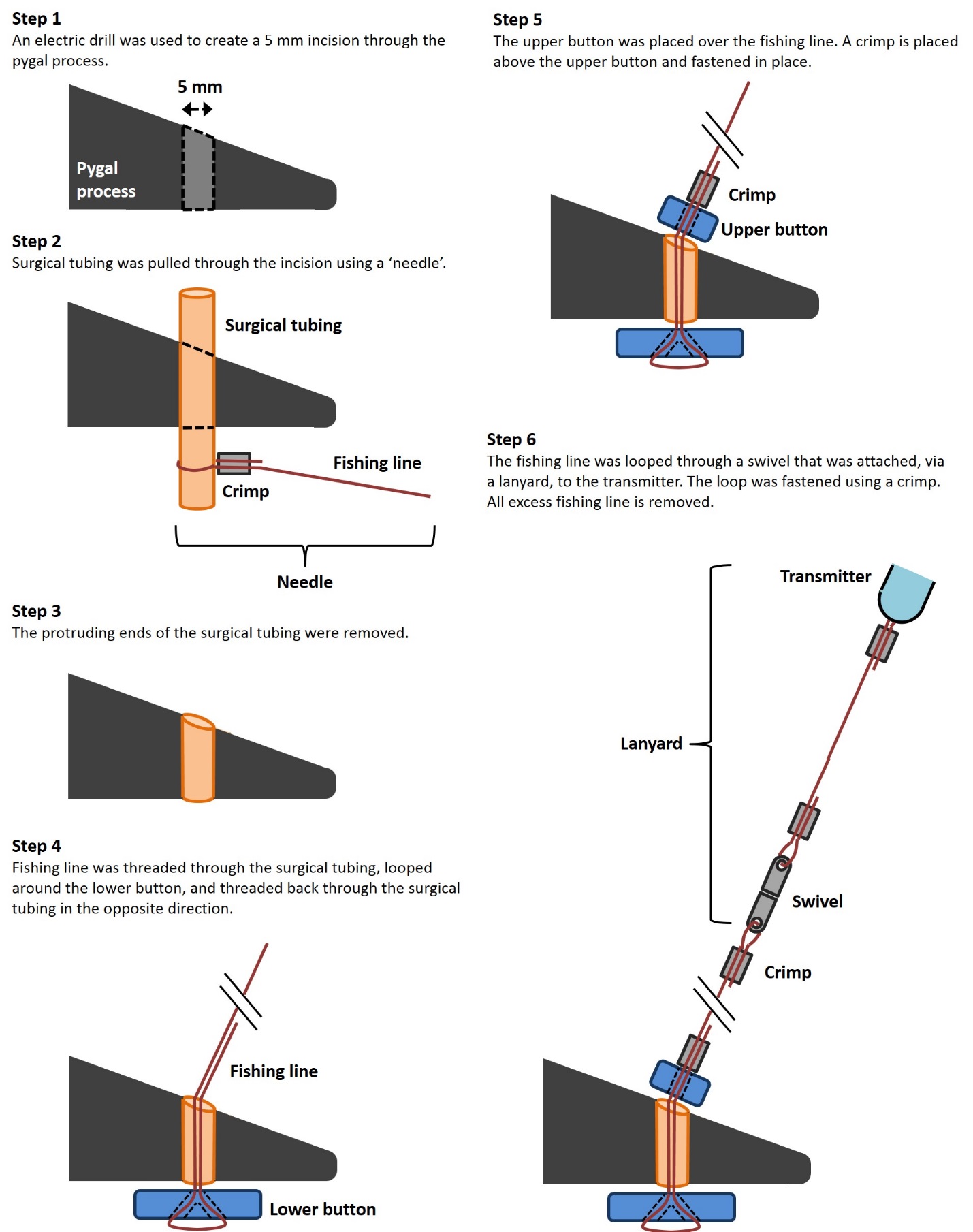
**

**Supplementary Figure 2**. Methods used in this study for the attachment of the PAT Mk10 transmitter onto nesting leatherback turtles *Dermochelys coriacea*.
